# gEVE: a genome-based endogenous viral element database provides comprehensive viral protein-coding sequences in mammalian genomes

**DOI:** 10.1093/database/baw087

**Published:** 2016-05-30

**Authors:** So Nakagawa, Mahoko Ueda Takahashi

**Affiliations:** ^1^Department of Molecular Life Science, Tokai University School of Medicine, 143 Shimokasuya, Isehara, Kanagawa 259-1193, Japan and; ^2^Micro/Nano Technology Center, Tokai University, 411 Kitakaname, Hiratsuka, Kanagawa, 259-1292, Japan

## Abstract

In mammals, approximately 10% of genome sequences correspond to endogenous viral elements (EVEs), which are derived from ancient viral infections of germ cells. Although most EVEs have been inactivated, some open reading frames (ORFs) of EVEs obtained functions in the hosts. However, EVE ORFs usually remain unannotated in the genomes, and no databases are available for EVE ORFs. To investigate the function and evolution of EVEs in mammalian genomes, we developed EVE ORF databases for 20 genomes of 19 mammalian species. A total of 736,771 non-overlapping EVE ORFs were identified and archived in a database named gEVE (http://geve.med.u-tokai.ac.jp). The gEVE database provides nucleotide and amino acid sequences, genomic loci and functional annotations of EVE ORFs for all 20 genomes. In analyzing RNA-seq data with the gEVE database, we successfully identified the expressed EVE genes, suggesting that the gEVE database facilitates studies of the genomic analyses of various mammalian species.

**Database URL:** http://geve.med.u-tokai.ac.jp

## Introduction

Approximately 10% of mammalian genome sequences correspond to endogenous viral elements (EVEs), including endogenous retroviruses (ERVs), which are thought to be derived from ancient viral infections of germ cells (1–4). In general, most EVEs have been inactivated by insertions, deletions, substitutions and/or epigenetic modifications. For this reason, they were once thought solely as the legacies of ancestral viral infection, so that they remain unannotated even if they contain open reading frames (ORFs). However, various ORFs of EVEs are still active and express viral proteins in hosts, some of which have been found to play important roles in mammalian development. For example, proteins that were originally derived from envelope proteins of retroviruses—many of them are called syncytins—are known to be involved in placental development in various mammalian species (5–16).

EVEs are unique in that their evolutionary histories differ among mammalian lineages. Various mammalian species have different syncytin genes that show similar molecular functions, but those have been acquired independently in each lineage during mammalian evolution (17, 18). For example, human syncytin-1 and -2 were captured in the ancestral lineages of Catarrhini and Simiiformes (6, 19), respectively and mouse syncytin-A and B were captured in the ancestral lineage of Muridae (7). Although this unique evolution of EVEs might have contributed to maintain genetic basis of mammalian traits, it is sometimes problematic for a comprehensive discovery of functional EVEs in mammalian genomes.

At present, there are no integrated databases of EVEs. Previously, EVE (ERV) databases for human and mouse genomes were constructed as HERVd (20) and ERE database (21), respectively. However, these databases have several problems (summarized in Table 1). For HERVd (http://herv.img.cas.cz), the reference human genome sequence is out of date, and the database is apparently not maintained, as its last update was on September 19, 2003. ERE database is not a web-based database and requires Microsoft Windows. Neither database provides ORFs for each EVE sequence. Further, no computational programs for EVE detection can identify EVE ORFs comprehensively in a given genome sequence. RetroTector (22) is a well-known computer program that can identify EVE sequences in a given genome sequence, but it has been reported to be unable to identify some EVE sequences (23). RepeatMasker (24) with Repbase (25) is another well-known system for detecting EVEs. However, it was originally developed as a ‘masking’ tool for repetitive sequences in a given genome, and cannot annotate ORFs originating from viruses. Although there are no established programs for EVE ORF detection, a combination of these programs and databases, as well as sequence similarity searches using endogenous and exogenous viral sequences, can be used to identify comprehensive sets of EVEs in a genome.

**Table 1.** Comparison of EVE databases.

Database (URL)	Species	Methods	Released date	Last update date	Reference
HERVd (http://herv.img.cas.cz)	Human	RepeatMasker with Repbase	Jul 2000	Sep 2003	20
ERE database (http://eredatabase.ucdmc.ucdavis.edu/)	Mouse	PCR library for LTR U3 sequences	Nov 2007	Feb 2008	21
		Homology search (Megablast)			
gEVE database (http://geve.med.u-tokai.ac.jp)	19 mammalian species	RetroTector	Apr 2014	Apr 2015	This paper
		RepeatMasker with Repbase			
		Homology search (BLAT)			

To investigate the function and evolution of EVEs in mammalian genomes, we developed a genome-based EVE database named gEVE (http://geve.med.u-tokai.ac.jp) using 20 genomes of 19 mammalian species (Table 2). We comprehensively identified and annotated EVE ORF sequences (i) encoding >80 amino acid (aa) sequences and (ii) harboring viral sequence motifs. The sequences and annotations of all EVEs can be downloaded from the database without registration. Our new annotations of EVE ORFs will offer a useful resource which enhances studies of EVEs, such as expression analysis using next-generation sequencing (NGS) data, facilitating studies of functional EVE sequences in various mammalian species.

**Table 2.** Genome data used in the gEVE database and EVE ORF viral profiles for each genome.

Species	Genome ID	Genome, released date	EVEs (Met)^a^	*gag*	*pro*	*pol* (LINE)^b^	*env*	others
Human (*Homo sapiens*)	Hsap38	GRCh38, Dec 2013	33 966 (31 292)	1782	1482	29 120 (21 087)	1731	11
Chimpanzee (*Pan troglodytes*)	Ptro214	CSAC 2.1.4, Feb 2011	30 099 (28 136)	1813	1125	25 572 (19 043)	1719	10
Gorilla (*Gorilla gorilla*)	Ggor31	gorGor3.1, May 2011	26 335 (24 409)	1456	1034	22 462 (16 140)	1486	8
Orangutan (*Pongo pygmaeus abelii*)	Pabe2	PPYG2, Sep 2007	28 315 (26 716)	1214	846	24 919 (19 492)	1400	14
Baboon (*Papio anubis*)	Panu2	Panu_2.0, Jun 2012	27 230 (25 192)	2101	1240	22 125 (15 476)	1962	5
Macaque (*Macaca mulatta*)	Mmul1	MMUL 1.0, Feb 2006	26 941 (25 043)	1980	1130	21 968 (15 745)	2020	7
Marmoset (*Callithrix jacchus*)	Cjac321	C_jacchus3.2.1, Jan 2010	21 802 (20 614)	992	406	19 575 (16 070)	888	3
Mouse (*Mus musculus*)	Mmus38	GRCm38.p1, Jan 2012	61 184 (58 805)	7494	5602	46 784 (29 122)	3075	16
Rat (*Rattus norvegicus*)	Rnor50	Rnor_5.0, Mar 2012	34 861 (32 525)	2570	1491	29 258 (21 517)	1771	6
Rabbit (*Oryctolagus cuniculus*)	Ocun2	oryCun2, Nov 2009	13 214 (12 909)	438	237	12 275 (10 473)	292	2
Cow (*Bos taurus*)	BtauUMD31	UMD3.1, Dec 2009	105 654 (104 674)	1023	673	103 402 (98 952)	648	1
Cow (*Bos taurus*)	Btau461	Btau_4.6.1 Nov 2011	98 016 (97 150)	860	641	96 065 (92 153)	585	0
Dog (*Canis lupus familiaris*)	Cfam31	CanFam3.1, Sep 2011	11 393 (11 011)	399	135	10 815 (10 019)	78	0
Cat (*Felis catus*)	Fcat62	Felis_catus_6.2, Sep 2011	11 132 (10 625)	694	203	9,898 (8,505)	391	1
Horse (*Equus caballus*)	Ecab2	EquCab2.0, Sep 2007	14 391 (13 972)	190	142	13 904 (12 554)	167	0
Sheep (*Ovis aries*)	Oari31	Oar_v3.1, Sep 2012	61 093 (60 184)	1099	517	58 940 (55 274)	628	1
Pig (*Sus scrofa*)	Sscr102	Sscrofa10.2, Aug 2011	15 210 (14 761)	456	155	14 350 (13 207)	285	9
Goat (*Capra hircus*)	Chir1	CHIR_1.0, Jan 2013	37 003 (36 060)	1106	508	34 797 (31 146)	653	0
Opossum (*Monodelphis domestica*)	Mdom5	monDom5, Oct 2006	77 190 (73 029)	2546	2723	71 821 (46 874)	1134	0
Platypus (*Ornithorhynchus anatinus*)	Oana5	OANA5, Dec 2005	1742 (1365)	2	1	1732 (1658)	7	0

a Number of EVE sequences containing at least an amino acid of Methionine was shown in parentheses.

b Number shown in parentheses indicates *pol* genes that were thought to be derived from LINEs, which were annotated as ‘LINE’ by RepeatMasker and/or ‘YP_073558.1’ or ‘NP_048132.1’ by BLASTP against the NCBI Viral Genome Database.

## gEVE database

### Statistics and annotation

**Figure 1.**
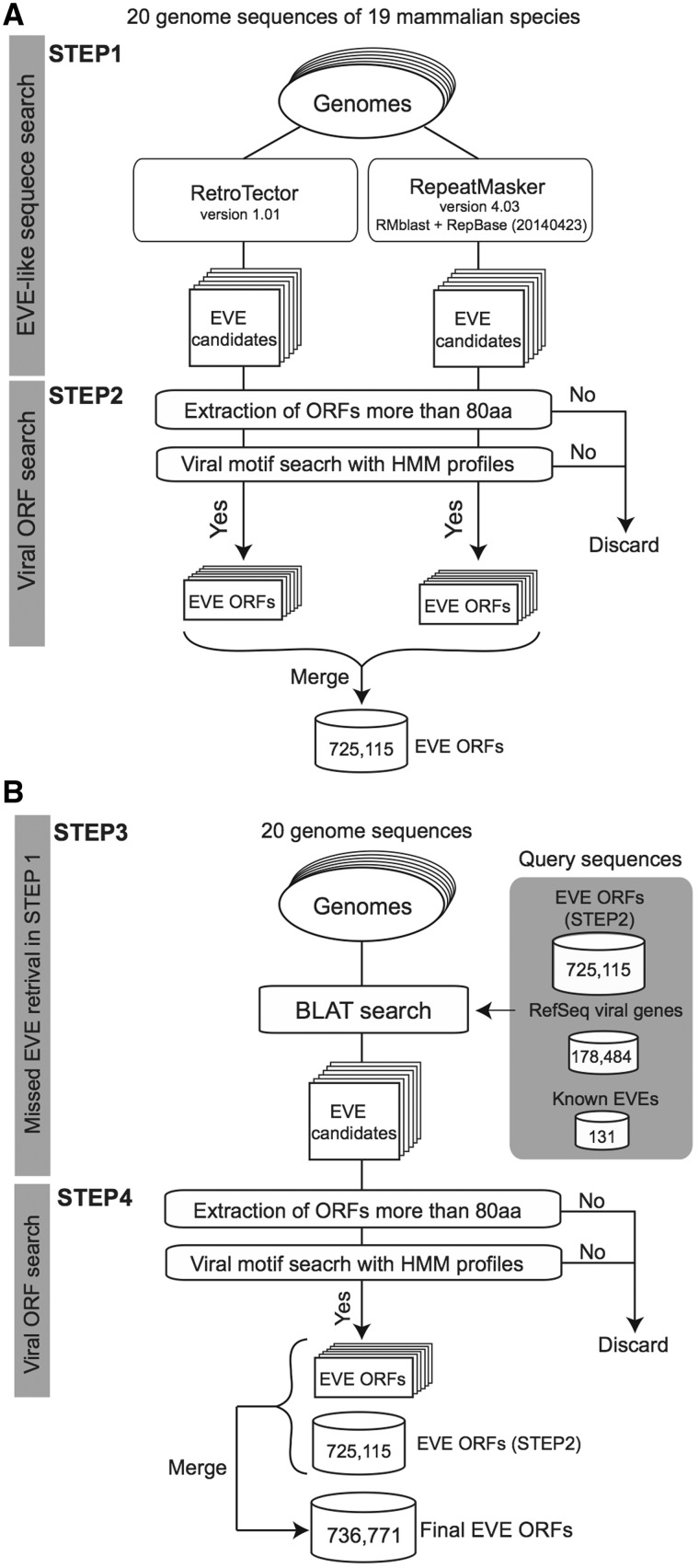
A schematic workflow of a four-step procedure for identifying EVE ORFs in 20 mammalian genomes. (**A**) First extraction of EVE candidates by RetroTector and RepeatMasker (STEP1) followed by ORF extraction processes in each genome (STEP2). (**B**) Second extraction of EVE ORFs by BLAT search for retrieving missed EVE candidates in STEP2 (STEP3). Similarly to the first extraction, EVE ORF datasets are generated by ORF extraction processes (STEP4). This is the final dataset of the gEVE database. The numbers for EVE ORF sequences in (A) and (B) indicate the total numbers of non-overlapping sequences in the 20 mammalian genomes. The numbers of extracted EVE sequences at STEP2 and STEP4 for each genome are shown in the Supplementary Table S3.

The procedure used to identify sequences derived from viral infection is summarized in Figure 1. We first applied RetroTector version 1.01 (22) and RepeatMasker version 4.03 (24) with RMblast (version 2.2.28) and RepBase (25, version 20140423) to each genome sequence (Figure 1A, STEP1). We used default parameters for each search program excluding RepeatMasker with the ‘-species’ option depending on the target genome: human, mouse, rat, cow, pig, cat, dog, or mammal. For each identified candidate region, we scanned all possible codon reading frames, three in each direction (i.e. six frames). If the longest reading frame in the region does not contain any stop codons encoding >80 amino acids (aa), the amino acid sequence was searched by using HMMER 3.1b1 (hmmer.org) with viral motif profiles as illustrated in Figure 1A STEP2. Hidden Markov models (HMMs) of the viral motif profiles used in this process were downloaded from the Pfam (26) and the Gypsy (27) databases (39 and 304 profiles, respectively, summarized in Supplementary Table S1). Each ORF having at least one HMM profile hit was stored in the database for the corresponding genome. Note that we used an arbitrary minimum ORF cut-off of 80 aa to reduce the number of falsely extracting non-coding RNAs as EVE ORFs (28). In our annotation, ORF sequences missing a start codon (ATG) are also defined as ORFs because these sequences could work as exons in a spliced transcript.

Next, to retrieve EVEs that are missed by the two computational programs, we performed similarity searches using BLAT (29) against each genome (Figure 1B, STEP3) using the following amino acid sequences: (i) all viral sequences encoding proteins stored in the NCBI RefSeq database (viral.1.protein.faa, version July 10, 2014), (ii) 131 known EVE genes (see Supplementary Table S2) and (iii) all 774 172 EVE sequences identified in the STEP 2. We then summarized EVE ORF sequences with viral motifs and encoding >80 amino acids by removing overlapping sequences while accounting for reading frames (Figure 1B, STEP 4). The number of EVE ORF sequences for each gene annotation is shown in Table 2 and the gEVE database (see ‘About’ page).

To further annotate each EVE ORF sequence, we conducted BLASTP searches separately against (i) all viral protein sequences (viral.1.protein.faa, version July 10, 2014), (ii) the non-redundant protein database (nr, version June 26, 2014) and (iii) known EVE sequences (see Supplementary Table S2). For each EVE gene, a description of the best hit was stored in the database. The number of best hits against all viral protein sequences for each genome is summarized in the gEVE database (see ‘About’ page). We also examined the correspondence between 131 known EVEs and sequences in the database (Supplementary Table S2 and ‘About’ page of the gEVE database). Additional annotations such as overlaps between exons of all annotated genes and our EVE sequences are provided in ‘Annotation Datasheet’ of the gEVE database. Detailed annotations are presented in the next section ‘Service and data download’.

In the database, we employed a naming system for each EVE ORF sequence based on the genome sequence and the EVE location, using a combination of genome ID, chromosome number, 5’ position, 3’ position and coordinates (+  or –). For example, a gEVE ID of Hsap38.chr1.100259758.100261128.– indicates that the EVE ORF is located on chromosome 1 of the human genome (version GRCh38) from positions 100 259 758 to 100 261 128 (on the negative strand). With this system, all EVEs have a unique ID for each genome.

### Service and data download

**Figure 2.**
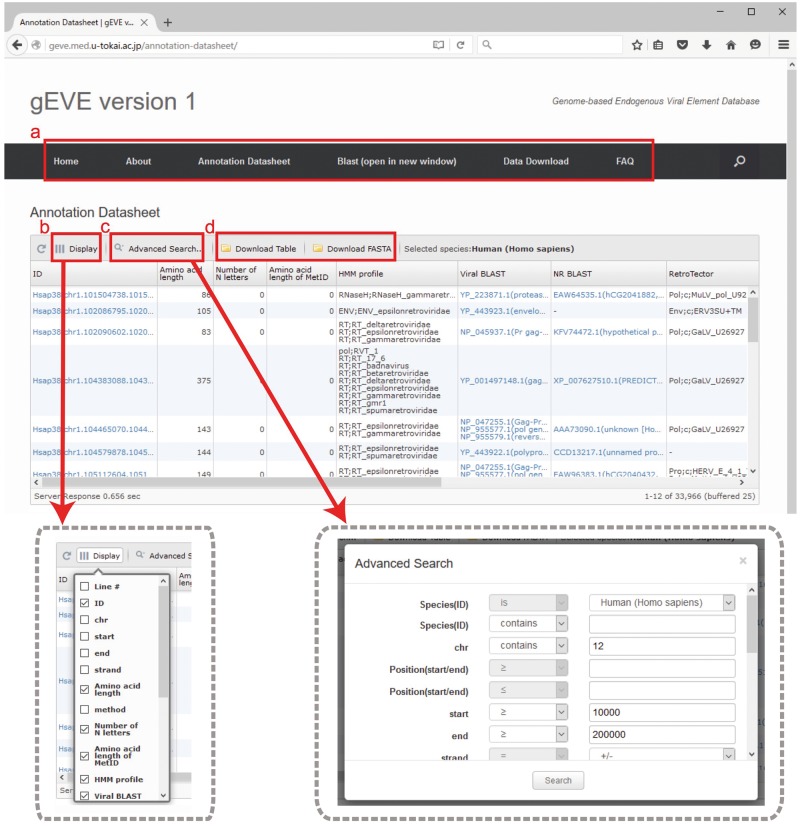
Web interface of the gEVE database. (**a**) A menu bar is shown at the top, and the current page is ‘Annotation Datasheet’. (**b**) Display option is available to select annotations of interest (boxed in gray dashed line, left). (**c**) Advanced searches for the EVE annotations such as genome IDs, viral HMM profiles, chromosome ID and amino acid lengths can be given in a new window (boxed in gray dashed line, right). (**d**) The annotation table or sequences (nucleotide and/or amino acid) shown in the window can be downloaded in tab-delimited format or FASTA format, respectively.

All EVE sequences and their annotations for the 20 mammalian genomes are available in the database. Annotation tables are displayed with optional searches (such as species, chromosomes, amino acid lengths and HMM profiles) and can be downloaded as tab-delimited text files (Figure 2). Annotation tables include the following information: ID, gEVE ID (genome ID, chromosome, start, end and strand); Amino acid length; method, method used for EVE identification; Number of N letters, the number of Ns (undetermined nucleotides) in the region; MetORF ID, ID for EVE starting with methionine; Amino acid length of MetORF ID; HMM profile, significant motif profile(s); Viral BLAST, BLASTP best hit(s) against the NCBI Viral Genome Database (viral.1.protein.faa, version 07/10, 2014); NR BLAST, BLASTP best hit(s) against the NCBI nr (non-redundant) database; and EVE BLAST, BLASTP best hit(s) against known EVE sequences; RetroTector, annotation by RetroTector (22); Repbase, annotation by RepeatMasker with Repbase database (24, 25); Overlapping, overlaps between EVE sequences and all annotated genes in the NCBI/UCSC/Ensembl databases. IDs, BLAST results and overlapping genes are linked to NCBI/UCSC/Ensembl resources depending on their contexts. Visible annotation column can be selected using ‘Display’ option (Figure 2b). Annotation search tools are also available (Figure 2c). FASTA files of nucleotide and/or amino acid sequences and annotation tables of selected EVE sequences can be downloaded via the website (Figure 2d). The bulk download of all the EVE ORF sequences and their annotations is available in the ‘Download’ page. Further, the BLAST search is implemented in the gEVE database powered by SequenceServer (30) so that any sequences of interest can be searched online against all sequences in the gEVE database.

### Application of the gEVE database

**Figure 3.**
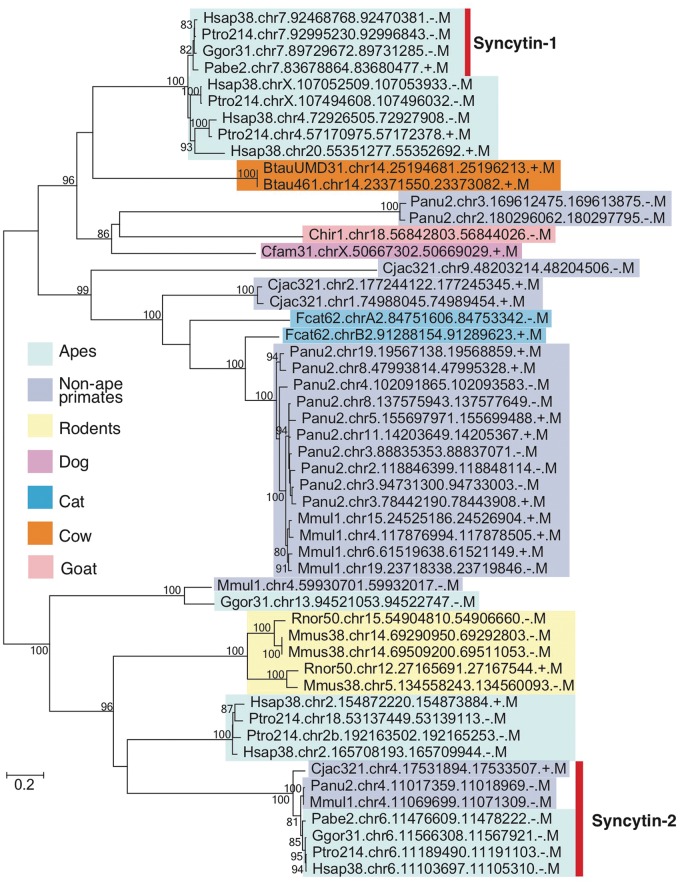
Phylogenetic tree of syncytin-1 like sequences. All sequences over 400 amino acids were extracted from BLASTP hits with e-values <e-40, and the tree was built with RAxML (31) with substitution model (JTT + G + I) determined by ProtTest3 (32). Bootstrap values are shown on the node (1,000 replicates). Known syncytin-1 and -2 genes in primates are indicated by the bar on the right. External nodes show EVE IDs (see Table 2 as well).

As described in Introduction, one of the difficulties in EVE analysis is the lack of conservation in sequences among mammalian lineages. We thus demonstrated phylogenetic analysis as an example of gEVE database application (Figure 3). Human syncytin-1 amino acid sequence was used to perform BLASTP searches against all EVE sequences in gEVE database with e-value <1e-40. Then, a maximum likelihood phylogenetic tree was constructed using RAxML version 8 (31). We obtained syncytin-1 genes in all apes as reported by Kim and his colleagues (19), and we also found syncytin-1 like sequences in non-hominid primates, rodents and even in cows, goats, dogs and cats. Interestingly, known annotated syncytin genes in cows, goats, dogs and cats are different from these syncytin-1 like sequences. This result does not directly indicate that all these syncytin-1 like sequences are really functional. However, we can easily know when these syncytin-1 like sequences were integrated in mammalian genomes. The phylogenetic analysis using gEVE database can help researchers to save time to obtain EVE ORFs in mammalian genomes and to select species for further comparative analysis.

The most powerful application of gEVE database is in NGS analyses. We also provide a General Transfer Format (GTF) file for EVE gene loci of each genome stored in the gEVE database (see ‘Download’ page). Using these GTF files with NGS data, dynamic expression profiles of EVE genes can be examined. For example, the RNA-seq data of human placenta expression (ID: ERR315374) stored in the sequence read archive (SRA, http://www.ncbi.nlm.nih.gov/sra/) were examined. The FASTQ sequences were obtained and mapped onto the human genome (GRCh38) using TopHat2 (33). The expression levels of EVE sequences were computed using Cufflinks (34) with the GTF file of gEVE Hsap38. The top 10 EVE sequences showing biggest FPKM values (i.e. highly expressed EVE sequences) are summarized in Table 3. We successfully identified known EVEs expressed in human placenta—PEG10 (35), suppressyn (10), syncytin-1 (5) and syncytin-2 (6)—as well as novel EVE sequences. This result shows that NGS data analyses combined with our annotation data enable us to discover hidden functional EVE sequences in genomes.

**Table 3.** Top 10 highly expressed gEVE sequences in the RNA-seq data of ERR315374

gEVE ID	HMM profile	Known EVE^a^	FPKM
Hsap38.chr7.94664474.94665679.+	*pro*	PEG10	481.4
Hsap38.chr7.94663299.94664531.+	*gag*	PEG10	392.9
Hsap38.chr3.129171078.129171320.-	*gag*	–	210.5
Hsap38.chr21.42917294.42917818.-	*env*	(suppressyn)	158.5
Hsap38.chr21.42918527.42919045.-	*env*	suppressyn	131.1
Hsap38.chr7.92468768.92470387.-	*env*	syncytin-1	44.5
Hsap38.chr21.42919026.42919586.-	*pol*	(suppressyn)	30.7
Hsap38.chr6.11103697.11105316.-	*env*	syncytin-2	24.6
Hsap38.chr21.42921853.42922110.-	*env*	(suppressyn)	24.2
Hsap38.chr16.20680984.20681253.+	*pol*	–	20.7

a A gene name in parentheses for a gEVE ID represents that the EVE sequence is located close to the known functional EVE sequence. A character, ‘–’, indicates the corresponding sequence is not reported to our knowledge.

### Future perspectives

We developed the gEVE database to provide EVE sequences coding >80 aa in the 20 mammalian genomes. In other words, our current database does not yet support non-coding sequences derived from EVEs. Accumulating reports indicate the functional importance of non-coding EVE sequences in host species, such as long terminal repeats (LTRs). Some LTRs in humans (such as LTR7) retain functional promoter–enhancer activity and control stem cell potency of embryonic stem (ES) and induced pluripotent stem (iPS) cells (36). Furthermore, various long non-coding (lnc) RNAs are expected to be derived from non-coding EVE sequences, which are also functional in host species (37). Thus, another task of gEVE database is to add more detailed annotation for EVE sequences. For example, evolutionary relationship among EVE sequences in the gEVE database has not been examined yet, although annotation of BLASTP best hits in the database would be partially useful. By addressing these points, the gEVE database will be continuously improved and expanded to contribute the further understanding of EVE sequences in the host genomes.

## Supplementary Data

Supplementary data are available at *Database* Online.

Supplementary Data
