# Supplementary material for: gEVE: a genome-based endogenous viral element database provides comprehensive viral protein-coding sequences in mammalian genomes
Source: Database (Oxford). 2016 May 30;2016:baw087. doi: 10.1093/database/baw087 (PMC4885607; doi:10.1093/database/baw087)
Supplement: Supplementary Data [file supp_2016_baw087_index.html]

gEVE: a genome-based endogenous viral element database provides comprehensive viral protein-coding sequences in mammalian genomes — Supplementary Data 

# gEVE: a genome-based endogenous viral element database provides comprehensive viral protein-coding sequences in mammalian genomes

## Supplementary Data

files

- Supplementary Data - xlsx file
